# Application of an Extracellular Matrix-Mimicking Fluorescent Polymer for the Detection of Proteolytic Venom Toxins

**DOI:** 10.3390/toxins15040294

**Published:** 2023-04-18

**Authors:** Eric Wachtel, Matyas A. Bittenbinder, Bas van de Velde, Julien Slagboom, Axel de Monts de Savasse, Luis L. Alonso, Nicholas R. Casewell, Freek J. Vonk, Jeroen Kool

**Affiliations:** 1 AIMMS, Division of BioAnalytical Chemistry, Department of Chemistry and Pharmaceutical Sciences, Faculty of Sciences, Vrije Universiteit Amsterdam, De Boelelaan 1085, 1081 HV Amsterdam, The Netherlands; 2 Centre for Analytical Sciences Amsterdam (CASA), 1098 XH Amsterdam, The Netherlands; 3 Naturalis Biodiversity Center, Darwinweg 2, 2333 CR Leiden, The Netherlands; 4 Centre for Snakebite Research & Interventions, Liverpool School of Tropical Medicine, Pembroke Place, Liverpool L3 5QA, UK; 5 Centre for Drugs and Diagnostics, Liverpool School of Tropical Medicine, Pembroke Place, Liverpool L3 5QA, UK

**Keywords:** snakebite, toxins, SVMP, extracellular matrix, cytotoxicity, tissue damage, high-throughput assay

## Abstract

The cytotoxicity caused by snake venoms is a serious medical problem that greatly contributes to the morbidity observed in snakebite patients. The cytotoxic components found in snake venoms belong to a variety of toxin classes and may cause cytotoxic effects by targeting a range of molecular structures, including cellular membranes, the extracellular matrix (ECM) and the cytoskeleton. Here, we present a high-throughput assay (384-well plate) that monitors ECM degradation by snake venom toxins via the application of fluorescent versions of model ECM substrates, specifically gelatin and collagen type I. Both crude venoms and fractionated toxins of a selection of medically relevant viperid and elapid species, separated via size-exclusion chromatography, were studied using the self-quenching, fluorescently labelled ECM–polymer substrates. The viperid venoms showed significantly higher proteolytic degradation when compared to elapid venoms, although the venoms with higher snake venom metalloproteinase content did not necessarily exhibit stronger substrate degradation than those with a lower one. Gelatin was generally more readily cleaved than collagen type I. In the viperid venoms, which were subjected to fractionation by SEC, two (*B. jararaca* and *C. rhodostoma*, respectively) or three (*E. ocellatus*) active proteases were identified. Therefore, the assay allows the study of proteolytic activity towards the ECM in vitro for crude and fractionated venoms.

## 1. Introduction

Snakebite envenoming is a serious health hazard and is listed as a Category A Neglected Tropical Disease (NTD) by the World Health Organization (WHO) [1,2]. An estimated 1.8–2.7 million snakebite cases occur annually, resulting in 81,000–138,000 fatalities and over 400,000 maimed victims. The regions that are most heavily affected include Southeast Asia, Sub-Saharan Africa, Latin America and Papua New Guinea, and snakebite disproportionally affects the rural, resource-poor communities in these regions [2,3,4,5,6]. Snake venoms consist of a broad range of protein families with numerous activities that may cause a variety of pharmacological effects. The pathophysiological effects caused by snake venoms can be broadly classified into neurotoxicity, haemotoxicity and cytotoxicity [2,5]. Cytotoxicity is the least studied of all venom pathologies and may cause local tissue damage (e.g., necrosis), (striated) muscle damage, rhabdomyolysis, microvascular damage, edema, blistering and pain [2,5,6,7,8,9,10]. 

Cytotoxicity can be caused by a variety of toxins, both enzymatic and non-enzymatic. Enzymatic cytotoxins include phospholipases A_2_ (PLA_2_s), snake venom metalloproteinases (SVMPs) and hyaluronidases, and non-enzymatic toxins comprise three-finger toxins (3FTxs), β-defensins, C-type lectins (CTL) or C-type lectin-like proteins (CLPs or snaclecs) and disintegrins [2,7,10,11,12]. Cytotoxins can either destabilize the cellular membrane, modify the cytoskeleton, induce oxidative stress or degrade the extracellular matrix (ECM) of cells [2,7,10,11,12,13,14,15]. This study will focus particularly on ECM-degrading toxins. The ECM is a macromolecular scaffold made up of proteoglycans, including hyaluronic acid, fibrous proteins (mainly collagens, elastins, fibronectins and laminins) and glycoproteins [10,16]. The ECM consists of the interstitial matrix, which mainly consists of collagen I, III, VI, XII and XIV, and the basement membrane (BM), which is made up of collagen type IV and VI, laminin, perlecan and nidogen [17,18]. The latter plays a key scaffolding role in capillary endothelial cells and other cell types [19]. 

Hyaluronidases and SVMPs are toxin classes capable of degrading ECM components [2,8,10,17,19,20]. The venoms of viperid snakes are particularly rich in SVMPs, while the SVMP content in most elapid snake venoms is considerably lower [21,22,23,24]. While commonly only a minor venom component, hyaluronidases are seemingly ubiquitous across snake venoms, though the relative abundance of this toxin class is higher in viperid venoms [8,25]. SVMPs are divided into three groups (P-I, P-II and P-III) based on the presence or absence of non-catalytic disintegrin/disintegrin-like or cysteine-rich domains in the C-terminal region [10,12,26]. The metalloproteinase domain is present in all three classes of SVMPs and hydrolyses key components of the ECM, mainly in the BM, including collagen IV, VI and XV, laminin, fibronectin, perlecan and BM-specific heparan sulfate proteoglycan (HSPG) [2,17,19]. This leads to the loss of integrity of the BM, weakening the scaffolding structure of endothelial cells in capillary vessels and therefore increasing the distensibility of the microvessel wall [19]. 

In order to study snake venom cytotoxicity, a variety of assays have previously been applied, ranging from in vivo wound exudate analysis by proteomics, immunochemical detection of ECM proteins in skin and exudates and various cell-based cytotoxicity assays to in vitro low-throughput assays such as zymography (utilizing non-fluorescent gelatin/collagen) and fluorometric assays assessing proteolytic activity with different fluorescently labelled peptide substrates (e.g., casein, rhodamine-110-peptide, desired peptide sequences) and self-quenched gelatin to assess protease activity [17,20,22,27,28,29,30,31,32,33,34,35,36]. These assays vary considerably in model complexity, specificity, throughput and costs.

Neumann et al. (2020) developed a generic high-throughput screening assay for profiling snake venom protease activity after high-resolution chromatographic fractionation, using rhodamine-110-peptide as a substrate, while Biardi et al. (2011) used self-quenched, fluorescently labelled gelatin to study SVMP activity in crude snake venom and the effects of inhibitors on this activity [27,34]. The goal of this study was to establish a similar assay using ‘true’ ECM components, harnessing a polymeric, self-quenched ECM-component- and ECM-mimicking compound (such as the self-quenched gelatin used by Biardi et al. 2011) to investigate the protease activity of both crude venoms and fractionated toxins. Two substrates were selected for use, namely fluorescently labelled gelatin and collagen type I, with the latter being the most relevant for studying venom-induced cytotoxicity by representing an ECM component. Both polymers are already being used to study non-venom proteases [37,38,39,40,41,42].

The assay utilizing the two self-quenched substrates was first optimized using crude venom. Next, venoms were separated by size utilizing size-exclusion chromatography (SEC), followed by nanofractionation analytics to assess the chromatographically separated and fractionated snake venom toxins for their potential in degrading the ECM substrates central to this study. Analytical SEC using a non-volatile phosphate buffer was performed, followed by characterization of the proteolytic compounds using a venomics approach from Slagboom et al. (2023) [43]. Our results demonstrated that the assay allowed the detection of ECM-degrading activity both in crude and fractionated venoms, as well as the potency of the respective venoms. The combination with nano-LC-MS/MS allowed the identification of the active compounds within the venoms. 

The protease assay presented here could prove valuable for better detecting and understanding venom-induced, cytotoxic pathologies caused by proteolytic components in snake venoms, which could be of importance for the process of improving snakebite treatments. 

## 2. Results and Discussion

We set out to establish high-throughput protease assays to enable the in vitro study of ECM-degrading venoms and the identification of venom toxins that mediate this activity. To do so, we used fluorescently labelled gelatin and collagen type I as substrates in 384-well plate assays. The assays developed were used to study both crude venoms and fractionated venom toxins.

### 2.1. Degradation of Gelatin and Collagen Type I by Crude Snake Venoms

**Figure 1:**
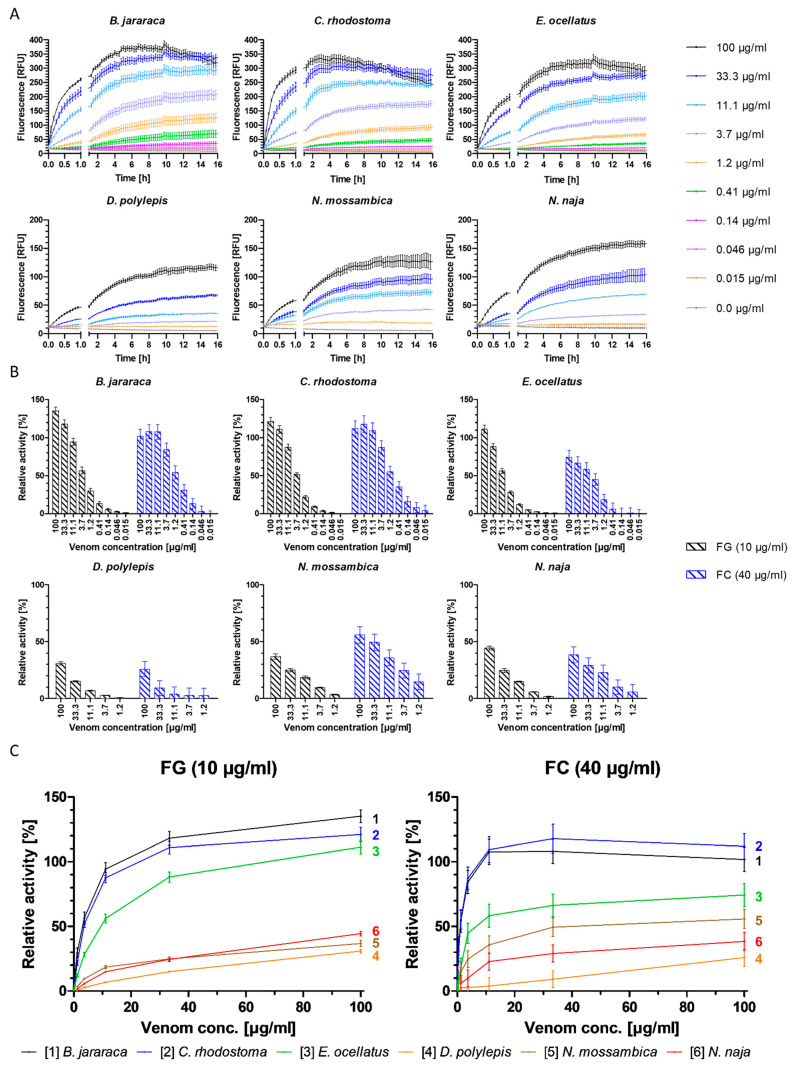
**Degradation of fluorescent gelatin and collagen type I by crude snake venoms and relative activity of venoms, normalized with collagenase type IV.** (**A**) The degradation of gelatin by snake venoms was monitored over 16 h, at 37 °C, at a substrate concentration of 10 µg/mL gelatin, with nine concentrations of viper venoms (100–0.015 µg/mL) or five concentrations of elapid venoms (100–1.2 µg/mL), compared to negative control (0.0 µg/mL). (**B**) Relative activity of snake venoms towards gelatin (FG) (black) and collagen (FC) (blue) normalized with positive control (100% activity); negative control was subtracted for baseline correction. Activity measured after five hours of incubation at 37 °C. (**C**) Direct comparison of relative activity between species of the six snake venoms towards gelatin (FG, left) and collagen I (FC, right), respectively. The three viper species show the highest relative activity compared to the elapid venoms for both substrates. Graphs are color-coded and numbered. Graphs are normalized with positive control (100% activity); negative control was subtracted for baseline correction. Activity measured after five hours of incubation at 37 °C. Each sample was measured in triplicate. Error bars show standard deviation.

The venoms of all six snake species included in this study degraded gelatin, a widely used, easily available and affordable standard polymer that mimics collagen. The three viperid venoms (*B. jararaca*, *C. rhodostoma* and *E. ocellatus*) were capable of cleaving gelatin at a high rate at venom concentrations of ≥33.3 µg/mL, even surpassing the proteolytic activity of the positive control (Figure 1, SI Figures S1–S3). For all viper venoms tested in this study, gelatin degradation detection limits were venom concentrations of ≥0.046 µg/mL (10 µg/mL gelatin), ≥0.14 µg/mL (5 µg/mL gelatin) and ≥0.41 µg/mL (2 µg/mL gelatin) (Figure 1 and SI Figure S1). A profound decrease in fluorescence (RFU) was observed at a venom concentration of 100 µg/mL for all gelatin and collagen I concentrations for the viper venoms after six to ten hours, indicating less cleavage (explanation see below). The elapid venoms (*D. polylepis*, *N. mossambica* and *N. naja*) cleaved gelatin at a lower rate than the viper venoms and the positive control (Figure 1, SI Figures S1–S3). The lowest concentration of 2 µg/mL gelatin failed to give satisfactory results regarding sensitivity (Figure 1 and SI Figure S1). 

Depending on the species, viper venom concentrations as low as 0.015–0.046 µg/mL at 10–40 µg/mL collagen I could be distinguished from the negative control (0.0 µg/mL) (SI Figure S2). As with gelatin, the high concentrations of viper venoms also gave a higher cleavage rate of collagen I than the positive control (SI Figures S2 and S3). For elapid venoms, a high concentration of collagen I (40 µg/mL) was required to achieve proper performance of the assay, and even with this substrate concentration, only tested elapid venom concentrations of 1.2 µg/mL and higher were distinguishable from the negative control (i.e., 11.1 µg/mL for *D. polylepis*, 3.7 µg/mL for *N. naja* and 1.2 µg/mL for *N. mossambica*) (SI Figure S2). As for gelatin, collagen I was degraded at a higher rate by the viper venoms than the elapid venoms. These experiments further revealed that, for collagen I, higher substrate concentrations (10–40 µg/mL) were needed than for gelatin (2–10 µg/mL) (Figure 1, SI Figures S1 and S2).

Overall, the higher the substrate concentration, the greater the separation of individual curves and overall higher fluorescence output, leading to higher sensitivity, which allowed for the more sensitive detection of the fluorescence increase as a result of substrate degradation (Figure 1, SI Figures S1 and S2). Fluorescent substrate concentrations of ≥2 µg/mL gelatin and ≥10 µg/mL collagen I for viper venoms and ≥5 µg/mL gelatin and ≥40 µg/mL collagen I for elapid venoms were selected as suitable substrate concentrations to ensure robust assay sensitivity. Fluorescence plateaued at all substrate and venom concentrations for both substrates after approximately five hours. Further, for some vipers, at the highest venom concentrations, decreases in fluorescence were observed after approximately five hours (Figure 1, SI Figures S1 and S2). This could be due to substrate depletion in combination with light-induced and/or chemical fluorescent product degradation, or due to buffer concentration increases resulting in a pH change caused by evaporation during the measurement (visual inspection of the plates post-measurement revealed reduced volumes in the wells). As fluorescein fluorescence properties are highly pH-dependent, this could lead to the observed fluorescence decrease [44]. Regardless, based on these combined observations, a measurement time of five hours was selected for use. 

Plotting the relative fluorescence (relative to the positive control) against the venom concentration allows the direct comparison of the cleavage activity of different venoms towards the respective substrate, and enables the identification of particularly potent venoms. The degradation of the two substrates varied significantly between both families for gelatin and collagen I, respectively (gelatin: t(0.995, 16) = 2.29, *p* = 1.81^−12^, Cohen’s d size = 7.61; collagen I: t(0.995, 16) = 2.29, *p* = 4.26^−6^, Cohen’s d size = 3.03). Both the viper and elapid venoms showed similar relative activity within their respective family, with viper venoms exhibiting higher overall activity (Figure 1 and Table 1). 

The necessity of a four-fold higher collagen I concentration than gelatin, in addition to higher relative activity towards gelatin, indicates that gelatin is more susceptible to degradation by these venoms than collagen type I, which is a finding supported by the literature [33]. The difference between collagen I and gelatin degradation was greatest in *E. ocellatus* and *B. jararaca* venom, followed by *N. naja* and *D. polylepis* venom. The difference in degradation of the two substrates is the smallest in *C. rhodostoma* venom, which shows an almost equally high cleavage rate for both substrates (Figure 1 and Table 1). One exception to this is that the venom of *N. mossambica* exhibited higher relative activity towards collagen I than gelatin (Figure 1 and Table 1). Collagens (e.g., collagen type I) are key structural proteins found in the ECM, as opposed to gelatin, which is thermally denatured or disintegrated collagen type I [45]. This denaturing by hydrolyzation leads to a loss of the triple-helix organization, which makes gelatin more susceptible to degradation by proteases, while collagen is more resistant and requires certain metalloproteinases, such as collagenases or SVMPs, for its degradation [17,20,45]. Collagen I therefore provides a better representation of the ECM-degrading potential of venoms, whereas gelatin could be a more suitable model for monitoring overall proteolytic activity [45]. 

The venoms of the three viper species included in this study are known to cause local tissue necrosis, edema and blistering, as well as systemic effects such as hemorrhage and coagulopathy. These are pathologies that are caused by SVMPs, among other toxins [5,17,46,47,48]. The venoms of the three viper species tested contain a relatively high abundance of SVMPs, with *E. ocellatus* venom being particularly rich in SVMPs (34.8–72.4%), with animals from Nigeria housed at LSTM containing 66.5% SVMPs in their venom according to Wagstaff et al. (2009) [23,47,49]. The venom of *C. rhodostoma* consists of 35.7–46.5% of SVMPs (venom from Thai specimens (Bangkok) contain 46.28% SVMPs in their venom, according to Tang et al. (2019)), while the venom of *B. jararaca* contains between 10.3% and 42.8%, depending on the locality [23,46,50,51,52,53]. Contrastingly, SVMPs are typically minor components of elapid venom, representing 0.9–16.2% of total toxins in *N. naja* venom, and 2.6% and 3.2% of the venom proteome of *N. mossambica* and *D. polylepis*, respectively [23,54,55,56,57,58,59,60]. These data seem likely to explain the lower degradation of gelatin and collagen I by the three elapid venoms compared to the viper venoms (Figure 1). 

The results above also indicate that venoms that are particularly rich in SVMPs might not necessarily have a higher gelatin and collagen I cleavage capacity compared to venoms with lower SVMP content within the respective family. As the locality of the snakes that the venoms were collected from is not known (with the exception of *E. ocellatus* and *C. rhodostoma*), it is unknown how high the actual SVMP share of the individual venoms is. It thereby remains inconclusive whether the SVMP abundance is the sole property affecting the activity detected in the developed bioassay. While there are clear distinctions between SVMP-rich viper and SVMP-deficient elapid venoms, data within these families indicate that overall higher SVMP content does not necessarily coincide with higher proteolytic activity (Figure 1 and SI Table S1), and therefore it is not possible to confirm with certainty if/how the SVMP content affects the venom potency in the assay. The ability of SVMPs to degrade ECM components is thought to be linked to their hemorrhagic activity, which varies between the SVMP subgroups (P-I, P-II and P-III) [5,19,20]. Correlating the presented in vitro data with actual ECM degradation in vivo remains challenging and therefore the assays presented in this study solely provide an indication of ECM degradation by snake venoms. 

### 2.2. Effects of Organic Solvents and Acidifiers on the Proteolytic Activity of Snake Venoms

Organic solvents and acidifiers are commonly used in HPLC, especially RP-HPLC. The effect of using an organic solvent (e.g., ACN) for SEC separation was compared to the non-organic buffer PBS, normally used for analytical SEC. Concentrations of up to 20% ACN did not have significant effects on the proteolytic activity of *E. ocellatus* venom. Concentrations higher than 20% resulted in a noticeable decrease in venom activity, with 50% ACN resulting in around half the activity of 0% ACN (SI Figure S4). PBS, even at higher concentrations (≥40%), had less impact on protein activity than ACN, with higher proteolytic activity remaining at all concentrations. At PBS concentrations above 80% and at longer assay run times (≥10 h), the activity started to decrease, although it was still not noticeably lower when compared to other PBS concentrations (SI Figure S4). Therefore, the inhibitory effect on the protein activity of PBS is negligible, especially with a 5 h assay runtime.

When the effects of the three acidifiers (e.g., FA, DFA and TFA) on the proteolytic activity of *E. ocellatus* venom were investigated, the outcomes were considerably different from those obtained with PBS. The presence of any of the three acidifiers tested resulted in a major decrease in protein activity at concentrations of ≥0.005%, indicating that a 0.1% acidifier concentration, often used in ‘conventional’ reversed-phase (and also in analytical SEC) HPLC (RP-HPLC) separations of proteins, would likely result in a strongly detrimental effect on the proteolytic venom activity, which also applied for ACN concentrations ≥30% (SI Figure S4). Completely excluding acidifiers from the mobile phase for RP-HPLC and SEC separations of venom into constituents resulted in very low separation quality (SI Figure S5). The observed decrease in activity in the presence of solvents and/or acidifiers is consistent with the observed effects in previous studies investigating protein stability when using volatile eluents [61,62,63]. Therefore, we chose to revert to an alternative SEC separation method with an eluent based on non-volatile salt buffers (see Section 4.4.2). This necessary approach comes at the cost of no direct MS compatibility.

### 2.3. Separation of Venoms Using Analytical SEC, followed by Bioactivity Profiling in Parallel with HT Venomics 

**Figure 2:**
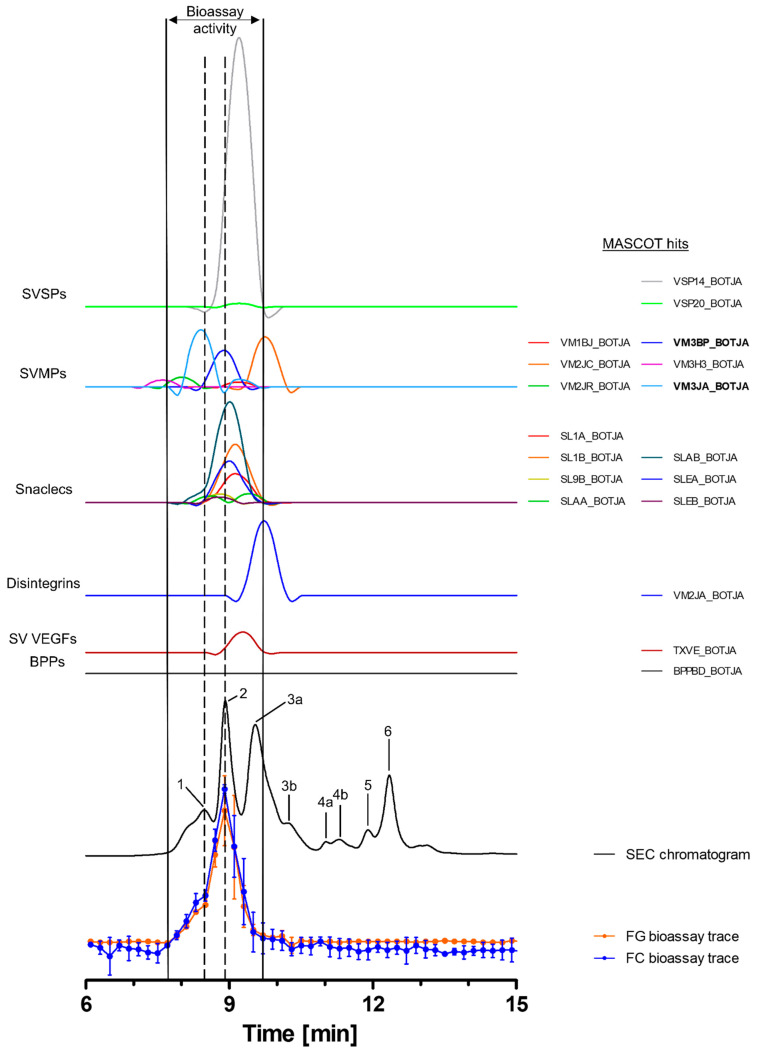
**Identification of gelatin- and collagen type I-degrading activity by proteolytic compounds in *B. jararaca* venom.** The upper graphs show the protein score chromatograms (PSCs), which represent the individual venom proteins found with Mascot database searching of the digested toxin content in the wells. The eluting toxins were detected at 220 nm, resulting in the SEC-UV chromatogram (black line). The lower two lines represent the bioassay traces (orange for gelatin (FG) and blue for collagen I (FC)). The vertical bold outer lines mark the area in which bioactivity has been detected and the vertical dashed lines mark the main activity peaks and their correlating SEC-UV chromatogram and PSC peaks. Bioactive toxins eluted between 7.8 and 9.7 min, with the main bioactivity peak corresponding to peak 1 and 2 in the SEC-UV chromatogram. In total, 18 toxins from six toxin classes were identified from the proteomics data (for which the corresponding information can be found in Table 2), with two toxins (jararhagin (VM3JA_BOTJA) and bothropasin (VM3BP_BOTJA)) being identified as the likely active compounds (highlighted with bold lettering). Bioactivity data were measured in triplicate; error bars show standard deviation.

**Figure 3:**
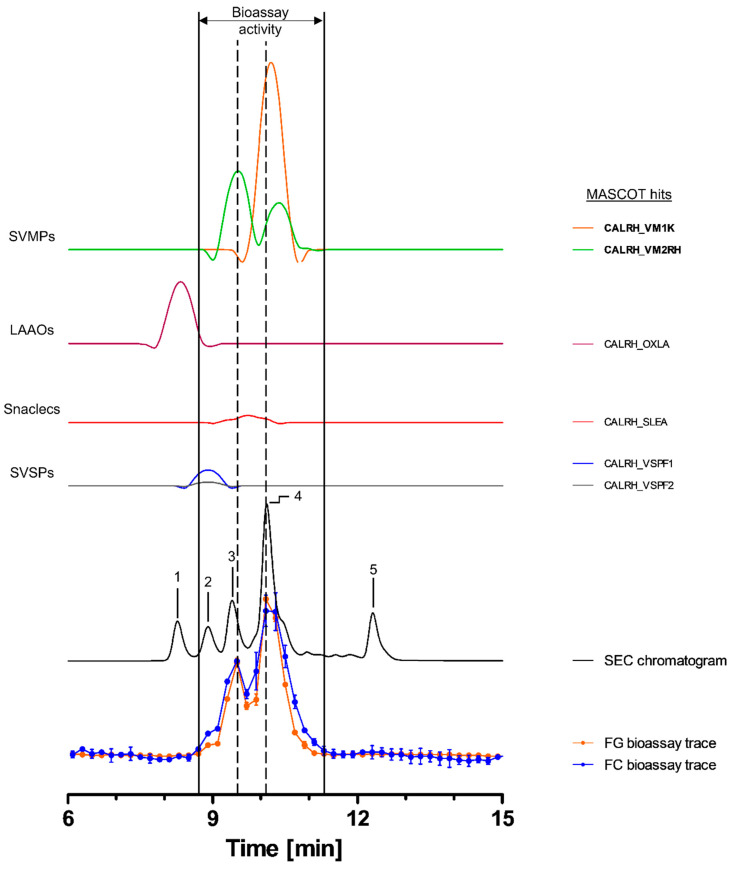
**Identification of gelatin- and collagen type I-degrading activity by proteolytic compounds in *C. rhodostoma* venom.** The upper graphs show the protein score chromatograms (PSCs), which represent the individual venom proteins found with Mascot database searching of the digested content in the wells. The eluting toxins were detected at 220 nm, resulting in the SEC chromatogram (black line). The lower two lines represent the bioassay traces (orange for gelatin (FG) and blue for collagen I (FC)). The vertical bold outer lines mark the area in which bioactivity has been detected and the vertical dashed lines mark the main activity peaks and their correlating SEC-UV chromatogram and PSC peaks. Bioactive toxins eluted between 8.7 and 11.1 min, with the bioactivity peaks corresponding to peaks 3 and 4 of the SEC chromatogram. In total, six toxins from four toxin classes were identified from the proteomics data (for which the corresponding information can be found in Table 2), with two toxins (kistomin (CALRH_VM1K) and rhodostoxin/disintegrin rhodostomin (CALRH_VM2RH)) being identified as the likely active compounds (highlighted with bold lettering). Bioactivity data were measured in triplicate. Error bars show standard deviation.

**Figure 4:**
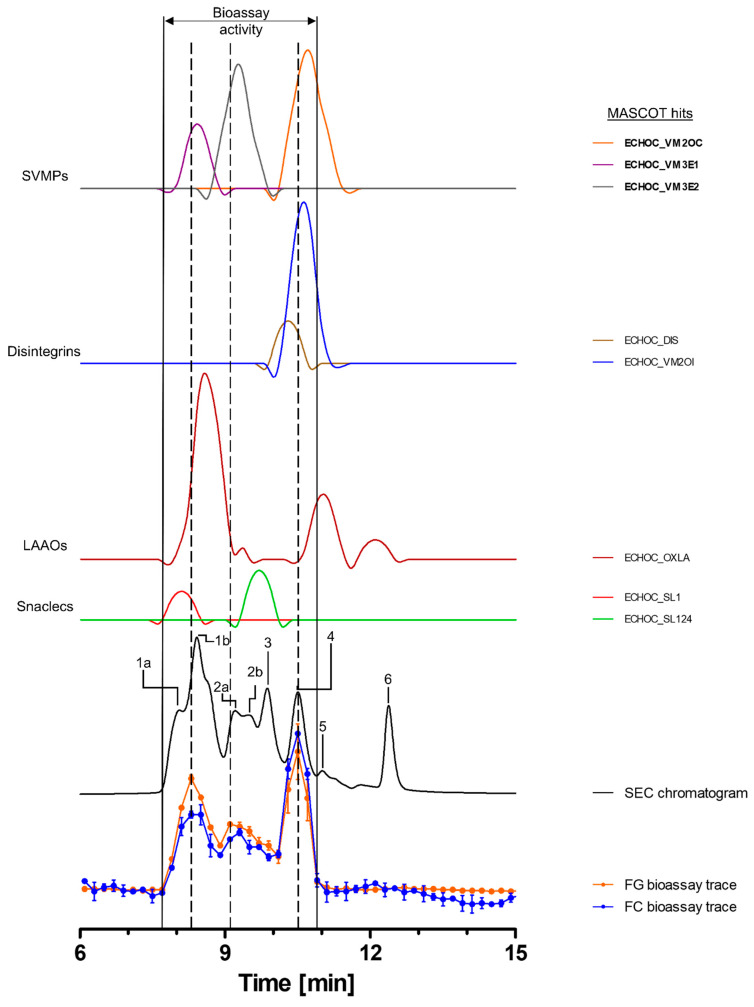
**Identification of gelatin- and collagen type I-degrading activity by proteolytic compounds in *E. ocellatus* venom.** The upper graphs show the protein score chromatograms (PSCs), which represent the individual venom proteins found with Mascot database searching of the digested content in the wells. The eluting toxins were detected at 220 nm, resulting in the SEC chromatogram (black line). The lower two lines represent the bioassay traces (orange for gelatin (FG) and blue for collagen I (FC)). The vertical bold outer lines mark the area in which bioactivity has been detected and the vertical dashed lines mark the main activity peaks and their correlating SEC-UV chromatogram and PSC peaks. Bioactive toxins eluted between 7.7 and 10.9 min, with two distinct and one broad bioactivity peak corresponding to peaks 1b and 4, plus peak 2a + b of the SEC chromatogram. Eight toxins from four different toxin classes were identified from the proteomics data (for which the corresponding information can be found in Table 2), with three toxins (Eoc1 (ECHOC_VM3E1), EoVMP2 (ECHOC_VM3E2) and Eoc6 (ECHOC_VM2OC)) being identified as the likely active compounds (highlighted with bold lettering). Bioactivity data were measured in triplicate. Error bars show standard deviation.

Analytical SEC with DPBS as the mobile phase and post-column fractionation was performed, as described in Section 4.4.2, to separate and collect venom toxins in their native forms. Actual toxin identification was performed in parallel to the post-column bioassays using the recently described HT venomics methodology by Slagboom et al. (2023) [43]. Gelatin and collagen I were used as the substrates to investigate its suitability to be used in post-separation bioassaying using nanofractionation analytics. For this, the three most proteolytically active venoms were used, namely *B. jararaca*, *C. rhodostoma* and *E. ocellatus.* Bioassay chromatograms for all three venoms were plotted using the post-column recorded bioassay data of the collected fractions (Figure 2, Figure 3 and Figure 4). 

#### 2.3.1. Bioassaying and Profiling of *B. jararaca* Venom after Analytical SEC 

Toxins in the venom of *B. jararaca* eluted over a time window between minute 7 and 14, with the SEC-UV chromatogram showing two smaller peaks (1 and 5), one small split peak (4a + b) and three major peaks (2, 3 and 6). Peak three is split into the main peak (3a) and a shoulder appearing after the main peak (3b). Peaks 1 and 5 eluted shortly before peaks 2 and 6 and partly co-eluted, respectively. Bioactive toxins eluted between 7.8 min and 9.7 min, with the main bioactivity peak corresponding to peak 2, and peak 1 matching the minor activity shortly before the main bioassay peak (Figure 2). This observation suggests at least two proteolytic compounds to be present in the venom. A total of 18 compounds belonging to six toxin classes were identified using the HT venomics approach. These included SVSPs (two toxins), SVMPs (two toxins and four toxin fragments), snaclecs (six toxin subunits and one subunit fragment), disintegrins (one toxin fragment), snake venom vascular endothelial growth factors (SV VEGFs; one toxin) and bradykinin-potentiating peptides (BPPs; one toxin) (Figure 2 and Table 2). Part of the bioassay peak profile overlapped well with the PSCs of the identified SVMPs and snaclecs. For the bioactivity peak profile, the PSC peaks for SVSPs and SV VEGFs overlapped partially (Figure 2). In terms of their proteolytic activity, SVMPs are known to degrade ECM components, indicating that they are responsible for the observed activity, as the other toxin classes identified are usually not associated with ECM degradation and therefore are unlikely to be responsible for the observed bioassay activity [2,20,32,64]. One specific P-III SVMP, namely zinc metalloproteinase-disintegrin-like bothropasin (VM3BP_BOTJA), correlated perfectly with the bioassay activity peak profile for both gelatin and for collagen I and peak 2 of the SEC chromatogram (Figure 2) [48]. Caseinolytic activity has previously been documented for this toxin [65]. A second P-III SVMP, zinc metalloproteinase-disintegrin-like jararhagin (fragment) (VM3JA_BOTJA), appeared to correlate in PSC peak shape and retention time with the shoulder of the bioactivity peaks seen in both the gelatin and collagen I assays, as well as with peak 1 of the SEC-UV chromatogram (Figure 2) [66,67]. As this toxin eluted before bothropasin, as deduced from the HT venomics data, it is likely responsible for the observed shoulder in the bioactivity peak. As jararhagin is known to hydrolyze collagen, amongst other ECM proteins, it is likely that it was responsible for the observed substrate degradation preceding the main bioassay peak (Figure 2) [67]. However, the main activity detected was most likely caused by bothropasin, as it correlated with the main bioassay peak, and it is therefore considered the most active ECM-degrading compound in *B. jararaca* venom, according to the presented bioassays. This also shows that not all SVMPs in the venom cleaved the two substrates, as only two of the six identified SVMPs exhibited this activity. This could be due to variations in the quantities of each of the SVMP toxins in the venom, with perhaps some eliciting insufficient substrate degradation activity or not showing proteolytic activity towards the substrates.

#### 2.3.2. Bioassaying and Profiling of *C. rhodostoma* Venom after Analytical SEC

Toxins in *C. rhodostoma* venom eluted between ~7.5 and 13.5 min, with the SEC-UV chromatogram showing one major (4) and four smaller peaks (1, 2, 3 and 5). Peaks 3 and 4 seemed to correlate with both peaks in the bioassay chromatograms (Figure 3). The bioactive toxins eluted between 8.7 and 11.1 min, with the main activity being recorded between 9.7 and 11.1 min (peak 4), while the first activity peak (8.7–9.7 min) exhibited a lower intensity (Figure 3). HT venomics identified a total of six compounds from four different toxin classes, including SVMPs (two toxins), SVSPs (two toxins), snaclecs (one toxin subunit) and L-amino-acid oxidases (LAAOs; one toxin). The two SVMPs, snake venom metalloproteinase kistomin (CALRH_VM1K) and zinc metalloproteinase rhodostoxin/disintegrin rhodostomin (CALRH_VM2RH), matched with the bioactivity peaks of both the substrates, correlating with peaks 3 (rhodostoxin/disintegrin rhodostomin) and 4 (kistomin and rhodostoxin/disintegrin rhodostomin) of the SEC-UV chromatogram (Figure 3 and Table 2). Kistomin is a P-I class SVMP and was found in the fractions causing the proteolytic activity corresponding to SEC-UV peak 4 only. This toxin has been shown to degrade fibrinogen and GP1b, as well as prolonging the latent period of platelet aggregation, blocking vWF-induced platelet activation and inhibiting the ATP secretion of human washed platelets [46]. The zinc metalloproteinase rhodostoxin/disintegrin rhodostomin was found in fractions aligning with both activity peaks (SEC peaks 3 and 4). While the disintegrin domain rhodostomin does not affect the proteolytic activity but is known to act as a potent platelet aggregation inhibitor, rhodostoxin, a P-II SVMP and the main hemorrhagic toxin in *C. rhodostoma* venom, is known to cause potent dermal hemorrhagic effects and has shown proteolytic activity [46,68]. On the upslope of the first bioassay peak, a small shoulder was visible, which correlated with peak 2 of the SEC-UV chromatogram. This peak overlapped with the two SVSP peaks thrombin-like enzyme ancrod and ancrod-2 (CALRH_VSPF1 and CALRH_VSPF2). They are known to be involved in venom-induced consumptive coagulopathy by catalyzing the release of fibrinopeptides from fibrinogen. Apparently, this proteolytic activity was picked up by our assays. It is important to note, however, that SVSPs are usually not associated with ECM degradation in vivo, but as they are proteases, they could express proteolytic activity in this assay [46,68,69]. From these data, it was summarized that most likely kistomin and rhodostoxin are the main toxins responsible for the proteolytic activity in *C. rhodostoma* venom, as deduced by the presented bioassays. 

#### 2.3.3. Bioassaying and Profiling of *E. ocellatus* Venom after Analytical SEC

Toxins in the venom of *E. ocellatus* eluted between 7 and 14 min, with four major peaks (1, 3, 4 and 6), one co-eluting twin peak (2a + b) and one smaller peak (5) observed in the SEC-UV chromatogram. Peak 1 was the largest peak, exhibiting a shoulder (1a) before the main peak (1b) (Figure 4). Bioactive toxins eluted from 7.7 to 10.9 min, with two distinct peaks between 7.7 and 8.9 min and 10.1 and 10.9 min and a third, broader peak around ~9–10 min. The first bioactivity peak correlated largely with peak 1b in the SEC-UV chromatogram, the second bioactivity peak with SEC-UV-peaks 2a and 2b, and the third peak in the bioassay chromatogram correlated with SEC-UV peak 4. In total, eight toxins, belonging to the toxin classes SVMPs (three toxins), disintegrins (two toxins), LAAOs (one toxin) and snaclecs (two toxins), were identified (Figure 4 and Table 2). Zinc metalloproteinase-disintegrin-like Eoc1 (ECHOC_VM3E1), a P-III SVMP, corresponding to peak 1b, has been shown to hydrolyze azocasein and the α- and β-chains of fibrinogen. Additionally, it inhibits endothelial cell adhesion in ECM proteins such as fibrinogen, fibronectin, vitronectin, collagen I and collagen IV [47,70]. Zinc metalloproteinase-disintegrin-like EoVMP2 (ECHOC_VM3E2) overlapped with peaks 2a and 2b from the SEC-UV chromatogram, and this P-III SVMP is known to exhibit strong hemorrhagic activity and inhibit collagen-induced platelet aggregation [71]. This hemorrhagic activity is based on the degradation of capillary basement membrane components such as collagen IV, laminin and fibronectin [71]. The final SVMP zinc metalloproteinase Eoc6/disintegrin ocellatusin (ECHOC_VM2OC) identified from the HT venomics data, which corresponded to SEC-UV peak 4, is a P-II SVMP consisting of a metalloproteinase and disintegrin domain [47,72]. The disintegrin domain ocellatusin, which is proteolytically processed, is known to inhibit platelet aggregation and is not considered to have proteolytic activity [47,72]. The zinc metalloproteinase Eoc6, however, is capable of impairing hemostasis in vivo and has been shown to have proteolytic activity [72]. The other toxins overlapping with these bioactivity peaks (disintegrins, snaclecs and LAAOs) are not known to exhibit proteolytic activity [2,10]. 

**Table 2:** **Toxins identified by nano-LC-MS/MS following tryptic digestion of fractionated toxins from the venoms of *B. jararaca*, *C. rhodostoma* and *E. ocellatus*.** Toxins identified as the bioactive compounds, which correlated with bioactive peaks observed in the bioactivity chromatograms from the gelatin and collagen I degradation bioassays, are marked in bold. Names and molecular weights (kDa) are according to the UniProt database (www.uniprot.org (accessed on 15 January 2023)). * Correlating SEC chromatogram peak (between: b/w). Remaining proteomics data are provided in Excel files in the Supplementary Information.

Species	Protein Class	Toxin	prot_acc	kDa	Peak *
*B. jararaca*	SVSP	Snake venom serine protease HS114	VSP14_BOTJA	27.834	3a
		Venom serine proteinase-like HS120	VSP20_BOTJA	27.815	3a
	SVMP	Snake venom metalloproteinase bothrojaractivase (Fragments)	VM1BJ_BOTJA	7.166	b/w 2 & 3a
		Zinc metalloproteinase/disintegrin (Fragment)	VM2JC_BOTJA	17.555	3a
		Zinc metalloproteinase-disintegrin jararin (Fragment)	VM2JR_BOTJA	16.835	before 1
		**Zinc metalloproteinase-disintegrin-like bothropasin**	**VM3BP_BOTJA**	**68.213**	**2**
		Zinc metalloproteinase-disintegrin-like HF3	VM3H3_BOTJA	67.695	before 1
		**Zinc metalloproteinase-disintegrin-like jararhagin (Fragment)**	**VM3JA_BOTJA**	**63.983**	**1**
	Snaclec	Snaclec bothrojaracin subunit alpha	SLAA_BOTJA	17.577	1 & 3a
		Snaclec bothrojaracin subunit beta	SLAB_BOTJA	17.292	2
		Snaclec botrocetin subunit alpha	SLEA_BOTJA	15.215	2
		Snaclec botrocetin subunit beta	SLEB_BOTJA	15.037	2
		Snaclec coagulation factor IX/factor X-binding protein subunit B (Fragment)	SL9B_BOTJA	3.582	2
		Snaclec GPIB-binding protein subunit alpha	SL1A_BOTJA	16.720	b/w 2 & 3a
		Snaclec GPIB-binding protein subunit beta	SL1B_BOTJA	14.298	b/w 2 & 3a
	Disintegrin	Disintegrin jarastatin (Fragment)	VM2JA_BOTJA	9.323	3a
	SV VEGF	Snake venom vascular endothelial growth factor toxin	TXVE_BOTJA	16.377	b/w 2 & 3a
	BPP	Bradykinin-potentiating peptide 11d	BPPBD_BOTJA	1.112	?
*C. rhodostoma*	SVSP	Thrombin-like enzyme ancrod	CALRH_VSPF1	26.570	2
		Thrombin-like enzyme ancrod-2	CALRH_VSPF2	29.145	2
	SVMP	**Snake venom metalloproteinase kistomin**	**CALRH_VM1K**	**47.446**	**4**
		**Zinc metalloproteinase rhodostoxin/disintegrin rhodostomin**	**CALRH_VM2RH**	**54.006**	**3 & 4**
	Snaclec	Snaclec rhodocetin subunit alpha	CALRH_SLEA	15.962	b/w 3 & 4
	LAAO	L-amino-acid oxidase	CALRH_OXLA	58.221	1
*E. ocellatus*	SVMP	**Zinc metalloproteinase Eoc6/disintegrin ocellatusin**	**ECHOC_VM2OC**	**55.165**	**4**
		**Zinc metalloproteinase-disintegrin-like Eoc1**	**ECHOC_VM3E1**	**68.751**	**1b**
		**Zinc metalloproteinase-disintegrin-like EoVMP2**	**ECHOC_VM3E2**	**69.426**	**2a & b**
	Snaclec	Snaclec 1	ECHOC_SL1	16.601	1a
		Snaclec CTL-Eoc124	ECHOC_SL124	16.882	2b & 3
	Disintegrin	Disintegrin ocellatusin	ECHOC_DIS	14.076	b/w 3 & 4
		Disintegrin ocellatin	ECHOC_VM2OI	5.526	4
	LAAO	L-amino-acid oxidase	ECHOC_OXLA	56.523	1b, 2a, 5 & 6

## 3. Conclusions 

The goal of this study was to develop and establish high-throughput screening assays that allow the high-throughput study of the proteolytic degradation of ECM substrates by (fractionated) snake venoms. Traditional zymography approaches using non-fluorescent gelatin and/or collagen as substrates, which are qualitative and semi-quantitative, and the 96-well-plate-based quantitative assay described by Biardi et al. (2011), are mainly suitable for low- to mid-throughput bioassays [27,73]. In order to validate the applicability of this high-throughput approach, the activity of a selection of medically relevant viperid and elapid venoms was investigated. For this, both crude venoms and fractionated toxins separated by analytical SEC were taken into account. Our approach using fluorescently labelled substrates allowed the post-column bioassaying of SEC-separated venom components, using only a low amount of venom sample and a low substrate concentration in a 384-well plate, making it a high-throughput method. Overall, the developed bioassays provide a framework for sensitive measurements of gelatin and collagen degradation, due to their fluorescein-labelled self-quenched conjugates, which yield highly fluorescent fragments when cleaved. The developed assay also allows the parallel identification of proteolytic venom components by the application of methodologies such as HT venomics. We foresee that the application of this bioassaying methodology could be valuable for a better understanding of venom-induced ECM-degrading effects and could be used in the process of developing new and improving existing snakebite treatments to better neutralize these toxin components that contribute to envenoming pathology. 

## 4. Material and Methods

### 4.1. Chemicals and Reagents

Water was purified to Milli-Q water grade using an in-house Milli-Q^®^ Reference Water Purification System (Millipore). Collagen–fluorescein (FITC) conjugate type I from bovine tendon was purchased from BioVision Inc. (Milpitas, CA, USA) The EnzChek™ gelatinase/collagenase assay kit, containing DQ^TM^ gelatin from pig skin fluorescein conjugate, was obtained from Invitrogen^TM^ by Thermo Fisher Scientific Inc. (Waltham, MA, USA) (lot 2,174,593 and 2,281,586). Gibco^TM^ Dulbecco’s phosphate-buffered saline (no calcium, no magnesium, pH 7.0 to 7.3; DPBS (2.67 mM KCl, 1.47 mM KH_2_PO_4_, 137.93 mM NaCl, 8.06 mM Na_2_HPO_4_-7H_2_O)) was purchased from Thermo Fisher Scientific Inc. Meanwhile, 2-propanol HPLC grade was purchased from VWR International. Acetonitrile HPLC-R (ACN), formic acid 99%, ULC/MS–CC/SFC (FA) and trifluoroacetic acid HPLC grade (TFA) were obtained from Biosolve B.V. Difluoroacetic acid for LC-MS (LiChropur, ≥97.5% (GC); DFA) was purchased from Sigma-Aldrich.

### 4.2. Venoms

Snake venoms were sourced from the venom library of the Faculty of Science, BioAnalytical Chemistry Division, Vrije Universiteit Amsterdam (VU). This library contains samples originally sourced from the Liverpool School of Tropical Medicine (LSTM), the National University of Singapore (NUS) and from captive breeders. The snake venoms used in this study came from the following viper (Viperidae) and elapid (Elapidae) species: *Bothrops jararaca* (jararaca, captive-bred), *Calloselasma rhodostoma* (Malayan pit viper, Thailand), *Dendroaspis polylepis* (black mamba, captive-bred), *Echis ocellatus* (West African carpet viper, Nigeria), *Naja mossambica* (Mozambique spitting cobra, captive-bred) and *Naja naja* (Indian cobra, captive-bred). Venoms from NUS were lyophilized immediately after milking, and then freeze-dried and stored at −80 °C. LSTM venoms were extracted, stored overnight at −20 °C and then lyophilized and stored at 4 °C for long-term use. Venoms extracted from snakes kept by private keepers were immediately flash-frozen and transported in liquid nitrogen, subsequently freeze-dried in the lab and stored at −80 °C. Samples were reconstituted in milli-Q water (mQ) to the desired stock solutions, depending on the type of assay. These solutions were then aliquoted and subsequently snap-frozen in liquid nitrogen and stored at −80 °C until use. All venoms were sourced prior to October 2014; therefore, these do not fall under the Nagoya Protocol [74]. 

### 4.3. High-Throughput Protease Assays to Study ECM Degradation by Crude Snake Venoms

The assays were based on a generic substrate degradation assay using fluorescently labelled gelatin (FG/gelatin) and collagen type I (FC/collagen I). Gelatin and collagen I were prepared as described by the manufacturer, protected from light and stored at −20 °C. Upon use, the substrates were diluted to the desired concentration with 1× reaction buffer. The 10× reaction buffer provided in the EnzChek™ assay kit (0.5 M Tris-HCl, 1.5 M NaCl, 50 mM CaCl_2_, 2 mM sodium azide, pH 7.6) was aliquoted and stored at −20 °C. Upon use, the buffer was diluted with sterile-filtered mQ to its final concentration. Collagenase type IV from *Clostridium histolyticum* (provided with the EnzChek™ gelatinase/collagenase assay kit) was used as a positive control at 1 U/mL, and was dissolved in sterile mQ and further diluted in 1x reaction buffer. The proteolytic properties of the venoms of *B. jararaca*, *C. rhodostoma* and *E. ocellatus* were investigated using three concentrations of gelatin (10, 5 and 2 µg/mL) and collagen I (40, 20 and 10 µg/mL). The venoms of *D. polylepis*, *N. mossambica* and *N. naja* were tested with identical concentrations of gelatin (10, 5 and 2 µg/mL) and a single concentration of collagen I (40 µg/mL). The venoms were selected to cover a range of medically significant species from the two snake families across a broad geographical range, covering regions greatly affected by snakebite. 

Assays were performed in clear 384-well flat-bottom microtiter plates (Greiner Bio One). Venoms were serially diluted in the well plate in reaction buffer: a 5-point dilution series was used for the elapid venoms (100; 33.3; 11.1; 3.7; 1.2 µg/mL) and a 9-point dilution series was performed with the viper venoms (100; 33.3; 11.1; 3.7; 1.2; 0.41; 0.14; 0.046; 0.015 µg/mL). Negative (45 µL 1x reaction buffer) and positive controls (40 µL 1× reaction buffer + 5 µL 10 U/mL collagenase solution) were included for each plate. Lastly, 5 µL of 10× gelatin or collagen I solution was added to the wells, resulting in a final well volume of 50 µL. 

Immediately after adding the substrate, the well plate was placed in a preheated (37 °C) Varioskan LUX Multimode Microplate Reader 3020-444 (ex. wavelength: 490 nm; em. wavelength: 525 nm), controlled by the SkanIt RE 4.1 software, and measured at 37 °C for 16 h (first hour: one measurement every five min; remaining measurement time: one measurement every 15 min; measurement time: 1 sec/well). Fluorescence was measured in relative fluorescence units (RFU). Each sample and all controls were performed in triplicate. As the assay principle was based on self-quenching substrates, the higher the fluorescence readout, the greater the substrate cleavage. In addition to plotting RFU against time, the activity of the venoms relative to the positive control (1 U/mL collagenase) at five hours was calculated with the following equation:

$$\frac{RFU\;\left(venom\right)-RFU\left(neg.\;ctrl.\right)}{RFU\;\left(pos.\;ctrl.\right)-RFU\;\left(neg.\;ctrl.\right)}*100=relative\;activity\;\left[\%\right]$$

Relative activity [%] was then plotted against the venom concentration [µg/mL]. The negative control was subtracted for baseline correction. An overview of the workflow is visualized in SI Figure S6A.

### 4.4. High-Throughput Protease Assays to Study ECM Degradation by Fractionated Snake Venom Toxins

In order to study the proteolytic components in our panel of snake venoms, we used analytical SEC in HPLC mode for toxin separation based on size. We employed SEC as a separation method to retain toxins in their native form and to avoid the potentially detrimental effects of separation on enzymatic protein activity. An overview of the complete workflow (SEC, fractionation, bioassay and proteomics) is visualized in SI Figure S6B.

#### 4.4.1. Effects of Organic Solvents, Buffers and Acidifiers on the Proteolytic Activity of Snake Venoms 

Volatile eluents and acidifiers are usually used in liquid chromatography, as they allow optimal protein separation and post-column removal without leaving high salt concentrations in wells, which might negatively affect bioassays, and would allow the direct coupling of SEC to MS, if desired. Previous studies have shown that the use of volatile eluents (e.g., using ACN or methanol as the organic modifier and acidifiers such as TFA) negatively affect protein stability, including labile proteases, by denaturing proteins through the destabilization of the tertiary and quaternary protein structures [61,62,63]. To investigate the negative effects of these components, we added *E. ocellatus* venom (5 mg/mL) to various concentrations of ACN (50, 40, 30, 20, 10, 5 and 0% in mQ) or the acidifiers FA, DFA or TFA (0.05, 0.01, 0.005, 0.001, 0.0005, 0.0001 and 0.0% in mQ, respectively) at a final venom concentration of 1 mg/mL. The venom was incubated at RT for 30 min, flash-frozen in liquid nitrogen and freeze-dried overnight. Upon use, the venom samples were reconstituted in 1x reaction buffer (final venom conc.: 1 mg/mL).

The incubation of *E. ocellatus* venom in PBS (80, 40, 20, 10, 5 and 0% in mQ) was performed by adding venom to the PBS solutions (final venom concentration: 1mg/mL) and it was incubated for 30 min at 21 °C (RT). These samples were not lyophilized and were added straight to the assay. All venom samples described above were subjected to the degradation assay with gelatin as a substrate, to assess their activity (final concentrations: 100 µg/mL of *E. ocellatus* venom and 10 µg/mL of gelatin). A positive (collagenase [1 U/mL]) and negative control were added to the assay plate and the assay performed as described in Section 4.3. 

#### 4.4.2. Separation of Venoms Using Size-Exclusion Chromatography 

The venoms of the species with the highest proteolytic activity (*B. jararaca, C. rhodostoma* and *E. ocellatus*) were fractionated using SEC on a Shimadzu HPLC system controlled with the Shimadzu Lab Solutions software. Venom stock solutions of 5 mg/mL were diluted with ice-cold mQ to a concentration of 2.5 mg/mL. Samples were injected with a Shimadzu SIL 20AC Prominence autosampler utilizing a 10 µL injection volume for venoms. Separation was performed on a Sepax Zenix SEC-300 column (300 Å, 5 µm, 4.6 mm × 300 mm), housed in a Shimadzu CTO-10AC VP column oven set to 27 °C. Solvent delivery was performed with a Shimadzu LC-10Ai pump set at a flow rate of 0.35 mL/min. The toxin elution from the column was monitored using a Shimadzu SPD-20A Prominence UV/Vis detector at 220 and 280 nm. The mobile phase consisted of 100% DPBS with isocratic elution over 20 min. A solvent loop was added to the pump system using a solvent mixture of 20% 2-propanol and 80% mQ, which was used between each run to flush the system and ensure proper toxin separation during subsequent runs. Following the SEC column, there was an analytical post-column adjustable flow splitter with a splitting ratio set to 1:9. From the total flow, 90% was directed to a fraction collector. Samples were collected as 12 sec fractions in clear 384-well flat-bottom microtiter plates (Greiner Bio-One) for a total of 18.0 min using a 6.0 min delay after the start of each run, resulting in 60 wells, each containing 62 µL collected eluent. For fractionation, a Gilson ASTED-XL autosampler rebuilt as a fraction collector was used, controlled by the Ariadne software (in-house written software, v1.08j). The plates were subsequently flash-frozen and stored at −20 °C until use. 

#### 4.4.3. Separation of Venoms Using Reversed-Phase HPLC

RP-HPLC separation was performed as described by Arrahman et al. (2022) with minor modifications [75]. Gradient separation was performed on a Waters XBridge Peptide BEH C18 column (300 Å, 5 μm, 4.6 mm × 100 mm, 1K-15K, 1/pk) housed in a Shimadzu CTO-10AC VP column oven set to 30 °C. Mobile phase A and B contained either 0.1% TFA or no acidifier, respectively. The venoms (2.5 mg/mL in mQ) of *E. ocellatus* and *N. mossambica* were separated utilizing the following gradients. *E. ocellatus*: Linear increase in solvent B from 0 to 30% over five minutes, followed by an increase from 30 to 50% of B in 25 min and a consecutive increase from 50 to 90% in four minutes, followed by 5-min isocratic elution at 90% B and a subsequent decrease to 0% B in one minute with a 10-min equilibration time at 0% B. *N. mossambica*: Linear increase in solvent B from 0 to 20% over five minutes, followed by an increase from 20 to 40% of B in 25 min. Solvent B was then increased from 40 to 90% in four minutes, followed by 5-min isocratic elution at 90% B and a subsequent decrease to 0% B in one minute with a 10-min equilibration time at 0% B. This set of experiments was performed to rule out RP-HPLC as a viable option for toxin separation and fractionation prior to the proposed bioassays.

#### 4.4.4. Degradation of Fluorescently Labelled Substrates by Fractionated Snake Venom Toxins Using the Protease Assays

After separation, the fractionated venom toxins were subjected to the protease assays under similar conditions as for the crude venoms (see Section 4.3), with minor modifications. Each well contained 35 µL of 1x reaction buffer, 10 µL of the respective venom fraction transferred from a plate with fractionated venom (in a selected time frame) and 5 µL gelatin or collagen I solution, resulting in a final substrate concentration of 10 µg/mL. Controls were added to each plate as described in Section 4.3. Plates were incubated for four hours at 37 °C, followed by measuring the plates in the preheated plate reader at 37 °C. For these analyses, end-point measurements (0.5 s/well) were performed (in triplicate) in order to reduce the plate reader measurement time needed per plate. 

### 4.5. High-Throughput In-Well Tryptic Digestion and nano-LC-MS/MS Analysis for Proteomics

In parallel with the bioactivity assays, following the analytical SEC separation, we performed proteomic analysis of the respective venom fractions. For this, we used the so-called ‘high-throughput venomics’ methodology recently described by Slagboom et al. (2023) [43]. After venom fractionation (see Section 4.4.2), 10 µL of sample from each well was pipetted into an empty 384-well plate. To each well, 25 µL of reduction buffer (25 mM ammonium bicarbonate and 0.05% β-mercaptoethanol; pH 8.2) was added using a pipetting robot (ThermoFisher Multidrop). The plates were then incubated at 95 °C for 15 min in the oven of a Hewlett Packard HP 6890 GC System and were allowed to cool down to RT, after which 10 µL of alkylating agent was added (12.5 mM Iodoacetamide) using the same Multidrop. After this step, the plates were incubated in the dark for 30 min at RT. A stock solution of trypsin (1 µg/µL in 50 mM acetic acid) was subsequently diluted 100 times in 25 mM ammonium bicarbonate to a concentration of 0.01 µg/µL, of which 10 µL was added using the Multidrop, and the plates were incubated overnight at 37 °C. The plates were centrifuged at 1000 rpm for 1 min in an Eppendorf Centrifuge 5810 R, followed by adding 10 µL of 1.25% formic acid to the plates. Finally, the plates were analyzed using nano-LC-MS/MS (or stored at −20 °C until analysis) as described by Slagboom et al. (2023) [43]. Venom samples were tryptically digested and then subjected to nano-LC separation on an UltiMate 3000 RSLCnano system (Thermo Fisher Scientific), followed by mass spectrometry. Mass spectrometry was performed on a maXis QTOF mass spectrometer (Bruker Daltonics) and the data processed using the Bruker DataAnalysis software, resulting in the identification of the proteins found in the individual venoms. Protein scores from each of the venom proteins were plotted against the corresponding retention times (*x*-axis) in order to generate so-called protein score chromatograms (PSCs) [43].

## Figures and Tables

**Table 1:** **Maximum relative degradation activity of venoms towards gelatin and collagen I (compared to Figure 1B).** Relative activity was calculated against positive control (equivalent to 100% activity). Error is in standard deviation.

**Viperidae**						
	*B. jararaca*		*C. rhodostoma*		*E. ocellatus*	
	gelatin	collagen I	gelatin	collagen I	gelatin	collagen I
Sub. conc. [µg/mL]	100	33.3	100	33.3	100	100
Max. rel. activity [%]	135.2 ± 4.9	108.1 ± 9.4	121.0 ± 5.5	117.8 ± 11.1	111.3 ± 5.1	74.3 ± 9.0
**Elapidae**						
	*D. polylepis*		*N. mossambica*		*N. naja*	
	gelatin	collagen I	gelatin	collagen I	gelatin	collagen I
Sub. conc. [µg/mL]	100	100	100	100	100	100
Max. rel. activity [%]	30.9 ± 1.4	25.9 ± 6.7	36.9 ± 2.3	55.9 ± 7.3	44.4 ± 1.7	38.5 ± 6.9

## Data Availability

Data is available upon request.

## Supplementary Materials

The following supporting information can be downloaded at: https://www.mdpi.com/article/10.3390/toxins15040294/s1, Table S1: Overview of the 6 species included in the study with the proportion, as deduced from the literature, of the twelve major toxin families in each venom (as percentage of total protein content in each venom). Abbreviations: PLA_2_, phospholipase A_2_; SVSP, snake venom serine protease; SVMP, snake venom metalloprotease; LAAO, L-amino acid oxidase; 3FTx, three-finger toxin; KUN, Kunitz peptides; CRiSP, Cysteine-Rich Secretory Protein; NP, natriuretic peptide; %WV, percentage of venom proteins identified (includes minor components not listed in table). Figure S1: Degradation of gelatin at substrate concentrations of 5 and 2 µg/mL by viper and elapid venoms: Cleavage of gelatin (FG) by B. jararaca, C. rhodostoma, E. ocellatus, D. polylepis, N. mossambica and N. naja venoms was monitored over 16 h, at 37 °C, at a substrate concentration of 5 µg/mL (A) and 2 µg/mL (B) FG with nine concentrations of viper venom (100–0.015 µg/mL) and five concentrations of elapid venom (100–1.2 µg/mL), compared to negative control (0.0 µg/mL). Each sample was measured in triplicate. Error bars show standard deviation. Figure S2: Degradation of collagen I at substrate concentrations of 40, 20 and 10 µg/mL by viper and elapid venoms: Cleavage of collagen I (FC) by B. jararaca, C. rhodostoma, E. ocellatus, D. polylepis, N. mossambica and N. naja venoms was monitored over 16 h, at 37 °C, at a substrate concentration of 40 µg/mL (A), 20 µg/mL (B) and 10 µg/mL (C) FC and nine concentrations of viper venom (100–0.015 µg/mL) and five concentrations of elapid venom (100–1.2 µg/mL), compared to negative control (0.0 µg/mL). Each sample was measured in triplicate. Error bars show standard deviation. Figure S3: Controls for the substrate degradation assays with gelatin and collagen I: Collagenase type IV from Clostridium histolyticum at 1 U/mL was used as the positive control. The negative control contained only buffer and the respective substrate at the respective concentration. A: Three gelatin concentrations were tested (2, 5 and 10 µg/mL FG). B: Three collagen I concentrations were tested (10, 20 and 40 µg/mL FC). Samples were each measured in triplicate. Error bars show standard deviation. Figure S4: Effects of different acidifiers (TFA, DFA and FA (0.05 to 0.0%)), a solvent (ACN (0–50%)) and a buffer (PBS (0–80%)) on the fluorescent gelatin degradation activity (10 µg/mL FG) of E. ocellatus venom (100 µg/mL): (A) Venom was incubated for 30 min at RT in the respective acidifiers at the listed concentrations in mQ. The assay was performed as described in Section 4.4. For all three acidifiers, the activity drastically decreased at additive concentrations of > 0.001%. Concentrations of ≤ 0.0005% did not affect the activity considerably. (B) Venom was incubated for 30 min at RT in the respective solvent or buffer at the listed concentrations mixed with mQ. The assay was performed as described in Section 4.4. Activity started to decrease considerably at 30% ACN, while high incubation concentrations (≥40%) of PBS had no considerable effect on venom activity and 80% PBS only led to a slight decrease in activity after >10 h of assay runtime. Measurements were performed in triplicate. Error bars show standard deviation. Figure S5: Separation of toxins by RP-HPLC: The venoms of E. ocellatus (A) and N. mossambica (B) were separated by RP-HPLC as described in Section 4.4.3 for the respective venoms, with 0.1% TFA as an acidifier (blue) or no acidifier (black), on the same mQ water–ACN gradient (upper and lower LC-UV chromatograms, respectively). The plotting of the chromatograms begins after the injection peak (3 min) and showcases the separation of the eluting toxins utilizing the two different solvent compositions. The absorbance of the chromatograms was measured at 220 nm. Figure S6: Graphical overview of the bioassay workflow: The workflow varies depending on whether it is used for crude (A) or fractionated venoms (B). (A) First, crude venom was serial-diluted in a clear 384-well flat-bottom plate in reaction buffer. Positive and negative controls were added to each plate. The DQ-quenched substrate was then added to the wells, resulting in the desired final substrate and venom concentration. The plate was placed into a plate reader and measured over five hours (ex. wavelength: 490 nm; em. wavelength: 525 nm; 1 s/well) at 37 °C and the fluorescence plotted against time. Each sample was measured in triplicate. (B) The desired venom was separated by SEC-HPLC and fractionated onto a clear 384-well flat-bottom plate. The plate was flash-frozen and stored at −20 °C. For the fractionated venom bioassay, 10 µL of each fraction was transferred to an individual well of a new 384-well flat-bottom plate with assay buffer, followed by addition of the DQ-quenched substrate. Plates were incubated for four hours at 37 °C, then transferred to a plate reader and measured (ex. wavelength: 490 nm; em. wavelength: 525 nm; 37 °C; 0.5 s/well). Activity was then plotted against the elution time of the respective well. For proteomics, 10 µL of each fraction was again transferred to an individual well of a new 384-well flat-bottom plate, followed by a tryptic digestion procedure directly in the well plate. Afterwards, the plate was directly transferred to nano-LC-MS/MS. nano-LC-MS/MS and the analysis of the resulting proteomics data were generated and analyzed as described by Slagboom et al. (2023) [43]. Created with BioRender.com.
