# Supplementary material for: Application of an Extracellular Matrix-Mimicking Fluorescent Polymer for the Detection of Proteolytic Venom Toxins
Source: Toxins (Basel). 2023 Apr 18;15(4):294. doi: 10.3390/toxins15040294 (PMC10143632; doi:10.3390/toxins15040294)
Supplement: Supplementary file 1 [file toxins-15-00294-s001.zip › toxins-2331822-supplementary.pdf]

toxins

Supplementary Materials: Application of an Extracellular Matrix-Mimicking Fluorescent Polymer for the Detection of Proteolytic Venom Toxins

Eric Wachtel, Matyas A. Bittenbinder, Bas van de Velde, Julien Slagboom, Axel de Monts de Savasse, Luis L. Alonso, Nicholas R. Casewell, Freek J. Vonk and Jeroen Kool

Supplementary Table S1. Overview of the 6 species included in the study with the proportion, as deduced from literature, of the twelve major toxin families in each venom (as percentage of total protein content in each venom). Abbreviations: PLA_2, phospholipase A_2; SVSP, snake venom serine protease; SVMP, snake venom metalloprotease; LAAO, L-amino acid oxidase; 3FTx, three-finger toxin; KUN, Kunitz peptides; CRiSP, Cysteine-Rich Secretory Protein; NP, natriuretic peptide; %WV, percentage of venom proteins identified (includes minor components not listed in table).

SPECIES	PLA_2	SVSP	SVMP	LAAO	3FTx	KUN	CTL/SNACLEC	DIS	CRiSP	NP	%WV	REF
B. jararaca	3.2 - 20.2	11.7 - 28.6	10.3 - 42.8	5.0 - 10.7			9.4 - 24.4	0.2 - 7.0	1.4 - 8.2	16.4 - 22.6	94.2 - 100	[1-4]
C. rhodostoma	4.4 - 13.8	13.6 - 21.3	35.7 - 46.5	7.0 - 11.1			10.2 - 26.3		2.0 - 2.6		96.0 - 100.0	[4-6]
E. ocellatus	8.5 - 21.2	1.7 - 15.5	34.8 - 72.4	1.4 - 2.2	0 - 2.2	0 – 1.5	4.0 - 7.0	2.0 - 6.8	0.3 - 2.9		93.5 - 99.6	[4, 7, 8]
D. polylepis	<0.1		3.2		31.0 - 40.0	39.0 - 61.1				2.9	95.3	[4, 9, 10]
N. mossambica	27.1		2.6		69.3						99	[11]
N. naja	0.0 -21.4	0 - 0.7	0.9 - 16.2	0 - 11.9	37.4 - 96.9	0 - 4.1			1.6 - 4.7	0 - 2.9	79.8 - 100.0	[4, 12-15]

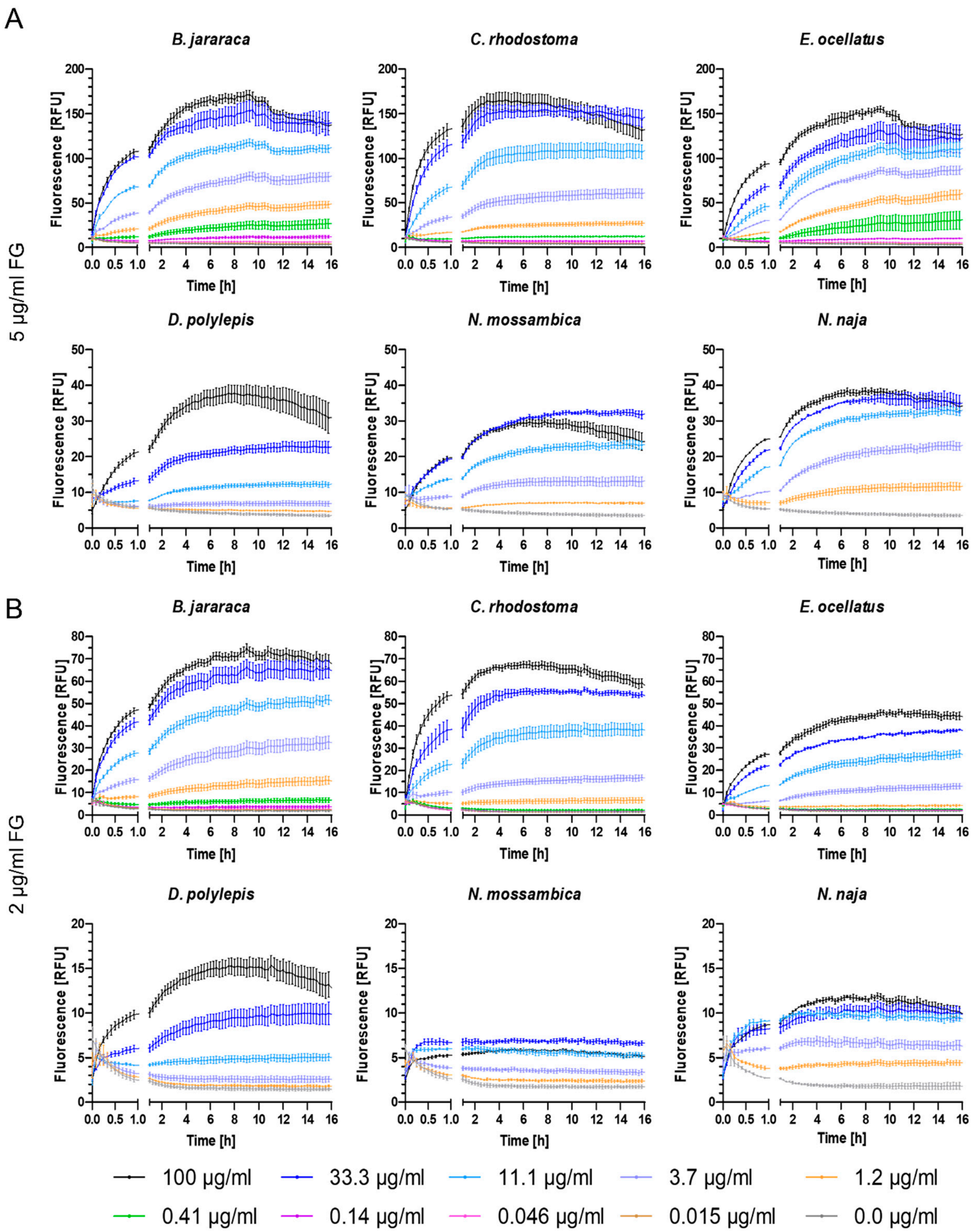

Supplementary Figure S1. Degradation of gelatin at substrate concentrations of 5 and 2 µg/ml by viper and elapid venoms: Cleavage of gelatin (FG) by *B. jararaca*, *C. rhodostoma*, *E. ocellatus*, *D. polylepis*, *N. mossambica* and *N. naja* venoms was monitored over 16 h, at 37°C at a substrate concentration of 5 µg/ml (A) and 2 µg/ml (B) FG with nine concentrations of viper venom (100 - 0.015 µg/ml) and five concentrations of elapid venom (100 - 1.2 µg/ml), compared to negative control (0.0 µg/ml). Each sample was measured in triplicate. Error bars show standard deviation.

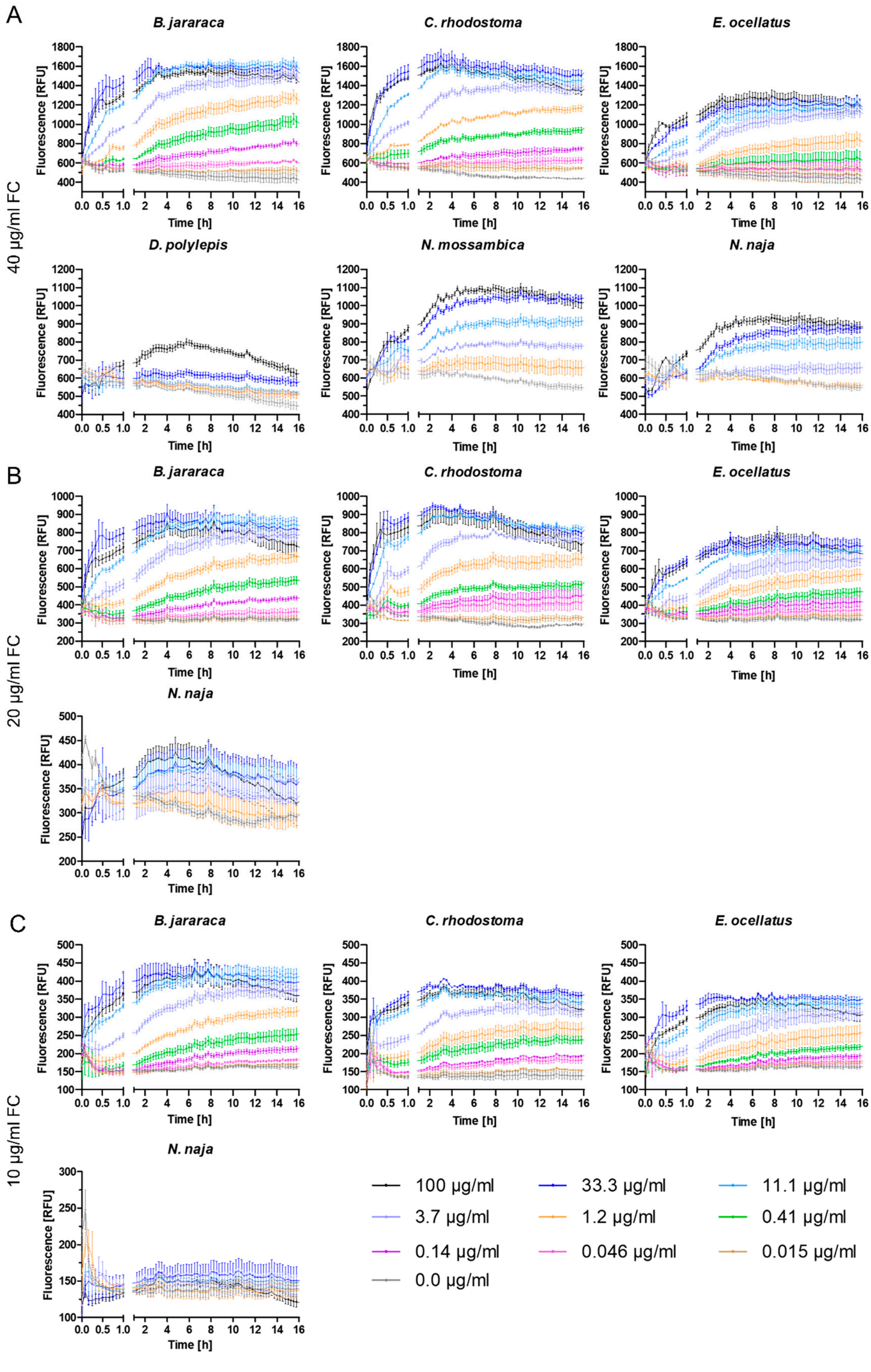

Supplementary Figure S2. Degradation of collagen I at substrate concentrations of 40, 20 and 10 µg/ml by viper and elapid venoms: Cleavage of collagen I (FC) by *B. jararaca*, *C. rhodostoma*, *E. ocellatus*, *D. polylepis*, *N. mossambica* and *N. naja* venoms was monitored over 16 h, at 37°C, at a substrate concentration of 40 µg/ml (A), 20 µg/ml (B) and 10 µg/ml (C) FC and nine concentrations of viper

venom (100 - 0.015 µg/ml) and five concentrations of elapid venom (100 - 1.2 µg/ml), compared to negative control (0.0 µg/ml). Each sample was measured in triplicate. Error bars show standard deviation.

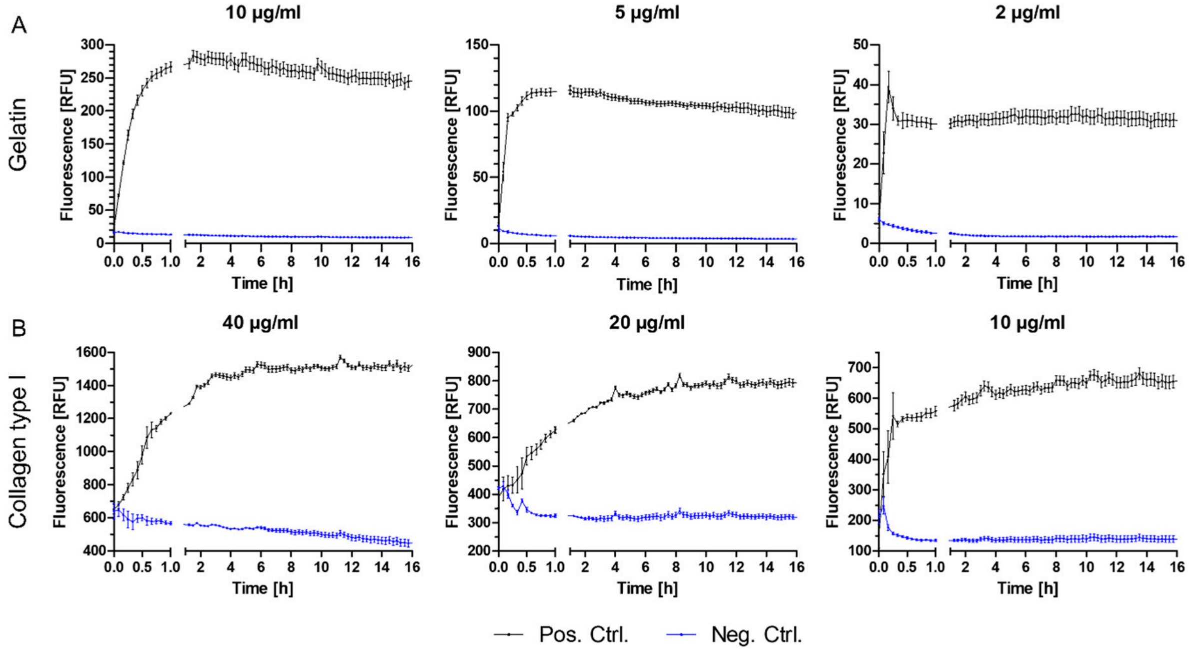

Supplementary Figure S3. Controls for the substrate degradation assays with gelatin and collagen I: Collagenase type IV from *Clostridium histolyticum* at 1 U/ml was used as the positive control. The negative control contained only buffer and the respective substrate at the respective concentration. A: Three gelatin concentrations were tested (2, 5 and 10 µg/ml FG). B: Three collagen I concentrations were tested (10, 20 and 40 µg/ml FC). Samples were each measured in triplicate. Error bars show standard deviation.

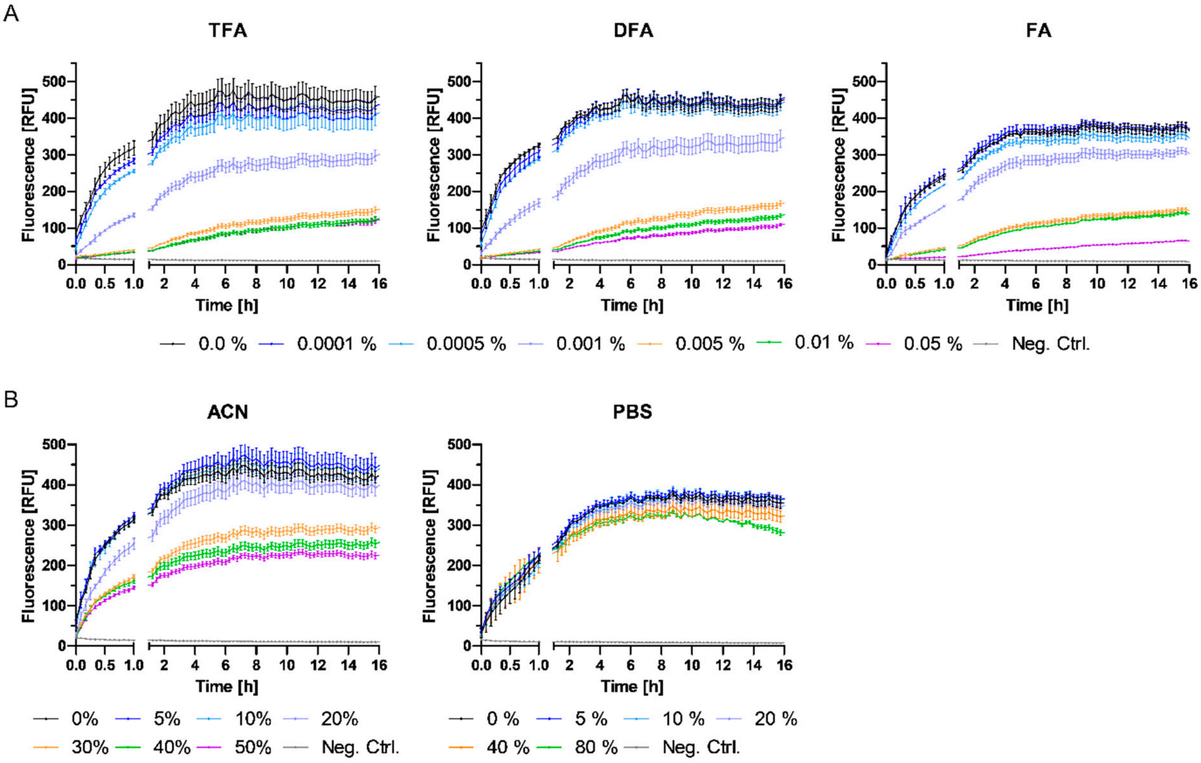

Supplementary Figure S4. Effects of different acidifiers (TFA, DFA and FA (0.05 to 0.0%)), a solvent (ACN (0 – 50%)) and a buffer (PBS (0 - 80%)) on the fluorescent gelatin degradation activity (10 µg/ml FG) of *E. ocellatus* venom (100 µg/ml): A) Venom was incubated for 30 min at RT in the respective acidifiers at the listed concentrations in mQ. The assay was performed as described in section 1.4. For all three acidifiers the activity drastically decreases at additive concentrations of > 0.001%. Concentrations of ≤ 0.0005% did not affect the activity considerably. B) Venom was incubated for 30 min at RT in the respective solvent or buffer at the listed concentrations mixed with mQ. The assay was performed as described in section 1.4. Activity starts to decrease considerably at 30%

ACN, while high incubation concentrations (≥40%) of PBS had no considerable effect on venom activity and 80% PBS only let to a slight decrease in activity after >10 h of assay runtime. Measurements were performed in triplicate. Error bars show standard deviation.

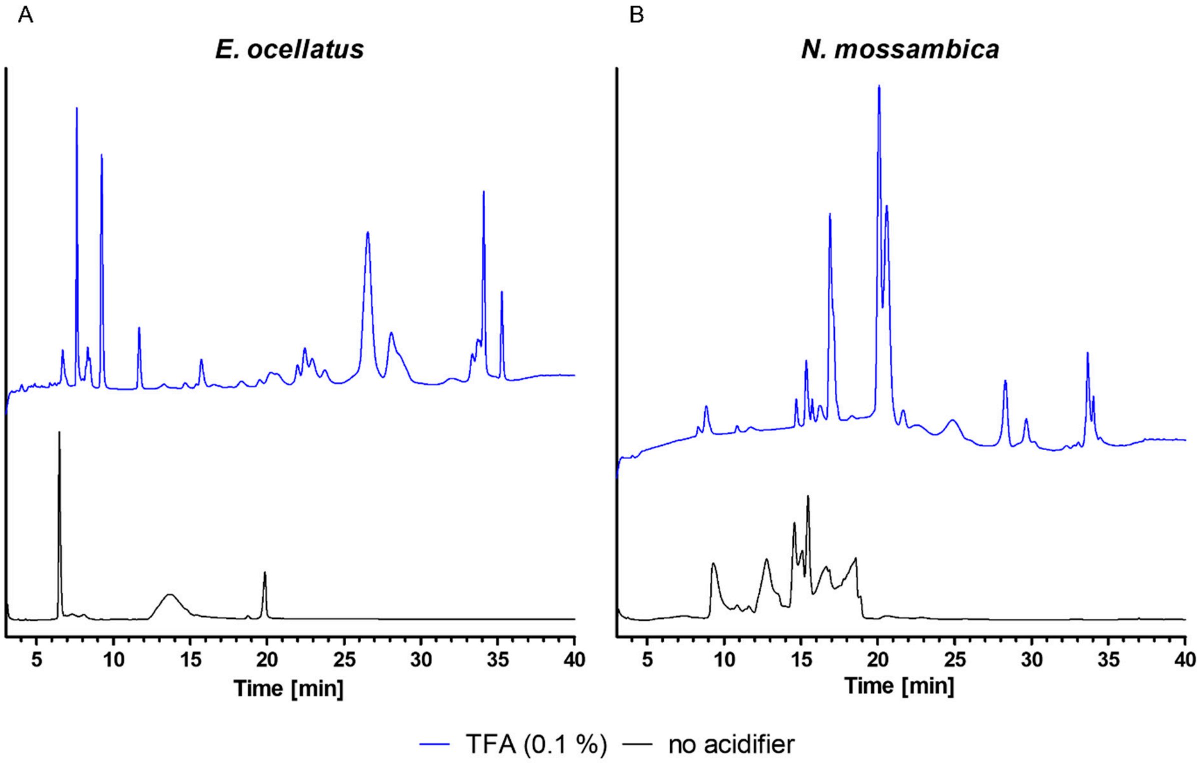

Supplementary Figure S5. Separation of toxins by RP-HPLC: The venoms of *E. ocellatus* (A) and *N. mossambica* (B) were separated by RP-HPLC as described in 1.4.3 for the respective venoms with 0.1% TFA as acidifier (blue) or no acidifier (black) on the same mQ water - ACN gradient (upper and lower LC-UV chromatograms, respectively). The plotting of the chromatograms begins after the injection peak (3 min) and showcases the separation of the eluting toxins utilising the two different solvent compositions. The absorbance of the chromatograms was measured at 220 nm.

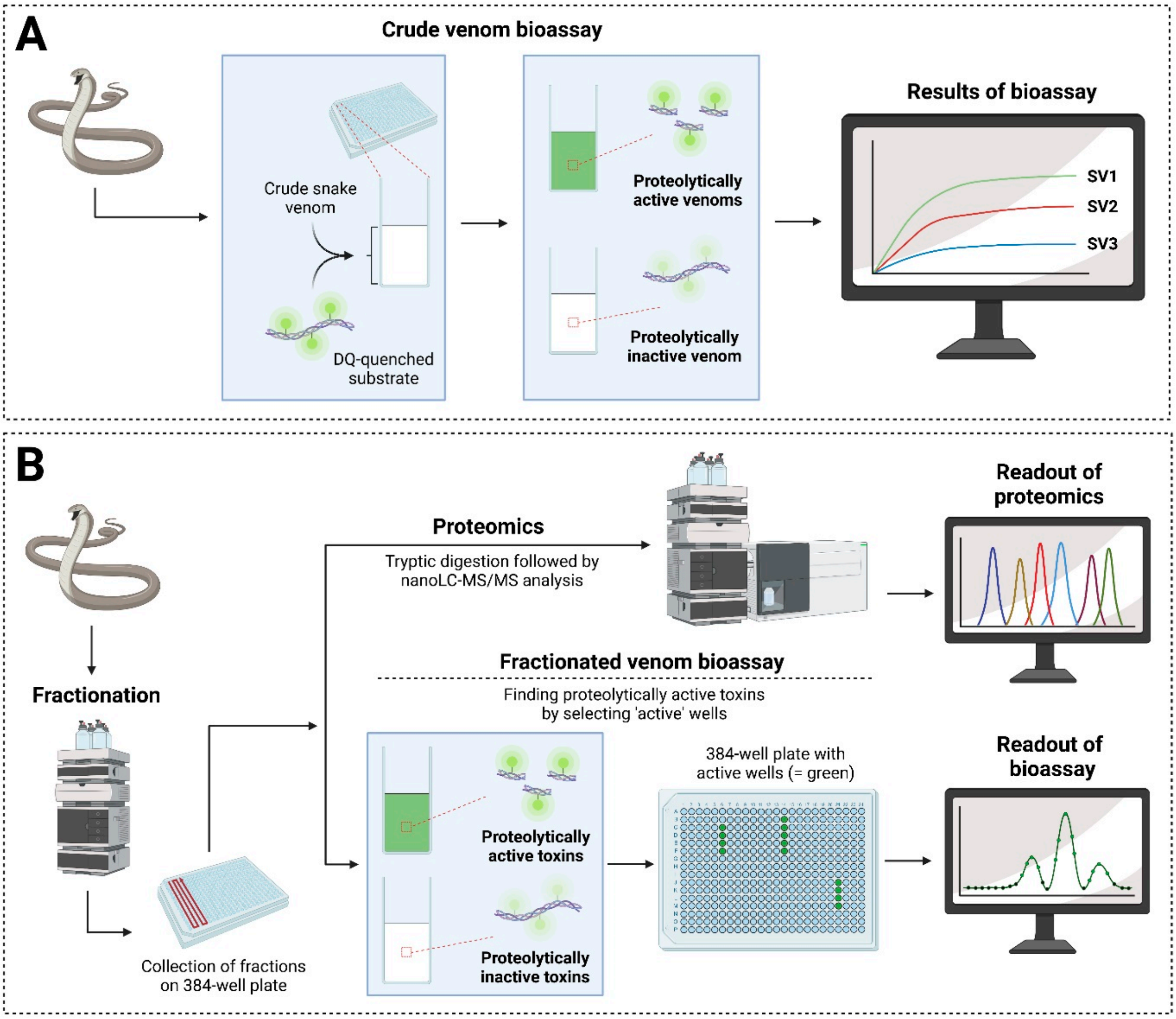

Supplementary Figure S6. Graphical overview of the bioassay workflow: The workflow varies, depending on if it is used for crude (A) or fractionated venoms (B). A) First, crude venom was serial-diluted in a clear 384 f-bottom well plate in reaction buffer. Positive and negative control were added to each plate. The DQ-quenched substrate was then added to the wells, resulting in a desired final substrate and venom concentration. The plate was placed into a plate reader and measured over five hours (ex. wavelength: 490 nm; em. wavelength: 525 nm; 1 s/well) at 37°C and the fluorescence plotted against time. Each sample was measured in triplicate. B) The desired venom was separated by SEC-HPLC and fractionated onto a clear 384 f-bottom well plate. The plate was flash-frozen and stored at -20°C. For the fractionated venom bioassay 10 µl of each fraction were transferred to an individual well of a new 384 f-bottom well plate with assay buffer, followed by addition of the DQ-quenched substrate. Plates were incubated for four hours at 37°C, then transferred to a plate reader and measured (ex. wavelength: 490 nm; em. wavelength: 525 nm; 37°C; 0.5 s/well). Activity was then plotted against the elution time of the respective well. For Proteomics 10 µl of each fraction were again transferred to an individual well of a new 384 f-bottom well plate, followed by a tryptic digestion procedure directly in the well plate. Afterwards the plate was directly transferred to nanoLC-MS/MS. nanoLC-MS/MS and the analysis of the resulting proteomics data was generated and analyzed as described by Slagboom et al. (2023) [16]. Created with BioRender.com.

References

1. Gonçalves-Machado, L., Pla, D., Sanz, L., Jorge, R. J. B., Leitão-De-Araújo, M., Alves, M. L. M., Alvares, D. J., De Miranda, J., Nowatzki, J. & de Morais-Zani, K. (2016) Combined venomics, venom gland transcriptomics, bioactivities, and antivenomics of two Bothrops jararaca populations from geographic isolated regions within the Brazilian Atlantic rainforest, *Journal of proteomics.* **135**, 73-89.
2. Nicolau, C. A., Carvalho, P. C., Junqueira-de-Azevedo, I. L., Teixeira-Ferreira, A., Junqueira, M., Perales, J., Neves-Ferreira, A. G. C. & Valente, R. H. (2017) An in-depth snake venom proteopeptidome characterization: Benchmarking Bothrops jararaca, *Journal of Proteomics.* **151**, 214-231.
3. Sousa, L. F., Nicolau, C. A., Peixoto, P. S., Bernardoni, J. L., Oliveira, S. S., Portes-Junior, J. A., Mourão, R. H. V., Lima-dos-Santos, I., Sano-Martins, I. S. & Chalkidis, H. M. (2013) Comparison of phylogeny, venom composition and neutralization by antivenom in diverse species of Bothrops complex, *PLoS neglected tropical diseases.* **7**, e2442.
4. Tasoulis, T. & Isbister, G. K. (2017) A review and database of snake venom proteomes, *Toxins.* **9**, 290.

5. Tang, E. L. H., Tan, C. H., Fung, S. Y. & Tan, N. H. (2016) Venomics of Calloselasma rhodostoma, the Malayan pit viper: A complex toxin arsenal unraveled, *Journal of proteomics.* **148**, 44-56.
6. Tang, E. L. H., Tan, N. H., Fung, S. Y. & Tan, C. H. (2019) Comparative proteomes, immunoreactivities and neutralization of procoagulant activities of Calloselasma rhodostoma (Malayan pit viper) venoms from four regions in Southeast Asia, *Toxicon.* **169**, 91-102.
7. Dingwoke, E. J., Adamude, F. A., Mohamed, G., Klein, A., Salihu, A., Abubakar, M. S. & Sallau, A. B. (2021) Venom proteomic analysis of medically important Nigerian viper Echis ocellatus and Bitis arietans snake species, *Biochemistry and Biophysics Reports.* **28**, 101164.
8. Wagstaff, S. C., Sanz, L., Juarez, P., Harrison, R. A. & Calvete, J. J. (2009) Combined snake venomics and venom gland transcriptomic analysis of the ocellated carpet viper, Echis ocellatus, *Journal of proteomics.* **71**, 609-623.
9. Ainsworth, S., Petras, D., Engmark, M., Süssmuth, R. D., Whiteley, G., Albulescu, L.-O., Kazandjian, T. D., Wagstaff, S. C., Rowley, P. & Wüster, W. (2018) The medical threat of mamba envenoming in sub-Saharan Africa revealed by genus-wide analysis of venom composition, toxicity and antivenomics profiling of available antivenoms, *Journal of proteomics.* **172**, 173-189.
10. Laustsen, A. H., Lomonte, B., Lohse, B., Fernández, J. & Gutiérrez, J. M. (2015) Unveiling the nature of black mamba (Dendroaspis polylepis) venom through venomics and antivenom immunoprofiling: Identification of key toxin targets for antivenom development, *Journal of proteomics.* **119**, 126-142.
11. Petras, D., Sanz, L., Segura, A., Herrera, M., Villalta, M., Solano, D., Vargas, M., León, G., Warrell, D. A. & Theakston, R. D. G. (2011) Snake venomics of African spitting cobras: toxin composition and assessment of congeneric cross-reactivity of the pan-African EchiTAb-Plus-ICP antivenom by antivenomics and neutralization approaches, *Journal of proteome research.* **10**, 1266-1280.
12. Chanda, A., Kalita, B., Patra, A., Senevirathne, W. D. S. T. & Mukherjee, A. K. (2019) Proteomic analysis and antivenomics study of Western India Naja naja venom: correlation between venom composition and clinical manifestations of cobra bite in this region, *Expert Review of Proteomics.* **16**, 171-184.
13. Chanda, A. & Mukherjee, A. K. (2020) Quantitative proteomics to reveal the composition of Southern India spectacled cobra (Naja naja) venom and its immunological cross-reactivity towards commercial antivenom, *International Journal of Biological Macromolecules.* **160**, 224-232.
14. Dutta, S., Chanda, A., Kalita, B., Islam, T., Patra, A. & Mukherjee, A. K. (2017) Proteomic analysis to unravel the complex venom proteome of eastern India Naja naja: Correlation of venom composition with its biochemical and pharmacological properties, *Journal of proteomics.* **156**, 29-39.
15. Senji Laxme, R., Attarde, S., Khochare, S., Suranse, V., Martin, G., Casewell, N. R., Whitaker, R. & Sunagar, K. (2021) Biogeographical venom variation in the Indian spectacled cobra (Naja naja) underscores the pressing need for pan-India efficacious snakebite therapy, *PLoS neglected tropical diseases.* **15**, e0009150.
16. Slagboom, J., Derks, R. J. E., Sadighi, R., Somsen, G., Ulens, C., Casewell, N. R. & Kool, J. (2023) High throughput venomics, *Journal of Proteome Research.* **Article ASAP**.
